# Autonomic dysfunction after mild acute ischemic stroke and six months after: a prospective observational cohort study

**DOI:** 10.1186/s12883-023-03054-4

**Published:** 2023-01-17

**Authors:** Mathias Damkjær, Sofie Amalie Simonsen, Adam Vittrup Heiberg, Jesper Mehlsen, Anders Sode West, Poul Jennum, Helle Klingenberg Iversen

**Affiliations:** 1 Clinical Stroke Research Unit, Department of Neurology, Rigshospitalet, University of Copenhagen, Valdemar Hansens Vej 1-23, 2600 Rigshospitalet, Denmark; 2 Section On Surgical Pathophysiology, Rigshospitalet, University of Copenhagen, Copenhagen, Denmark; 3 Danish Center for Sleep Medicine, Department of Neurophysiology, Rigshospitalet, University of Copenhagen, Copenhagen, Denmark; 4 Faculty of Health and Medical Sciences, University of Copenhagen, Copenhagen, Denmark

**Keywords:** Stroke, Autonomic Dysfunction, Tilt-table test, Valsalva maneuver, Heart rate response to deep breathing, Active standing, Catecholamines

## Abstract

**Introduction:**

Autonomic dysfunction is prevalent in ischemic stroke patients and associated with a worse clinical outcome. We aimed to evaluate autonomic dysfunction over time and the tolerability of the head-up tilt table test in an acute stroke setting to optimize patient care.

**Patients and method:**

In a prospective observational cohort study, patients were consecutively recruited from an acute stroke unit. The patients underwent heart rate and blood pressure analysis during the Valsalva maneuver, deep breathing, active standing, and head-up tilt table test if active standing was tolerated. In addition, heart rate variability and catecholamines were measured. All tests were performed within seven days after index ischemic stroke and repeated at six months follow-up.

**Results:**

The cohort was comprised of 91 acute stroke patients, mean (SD) age 66 (11) years, median (IQR) initial National Institute of Health Stroke Scale 2 (1–4) and modified Ranking Scale 2 (1–3). The head-up tilt table test revealed 7 patients (10%) with orthostatic hypotension. The examination was terminated before it was completed in 15%, but none developed neurological symptoms. In the acute state the prevalence of autonomic dysfunction varied between 10–100% depending on the test. No changes were found in presence and severity of autonomic dysfunction over time.

**Conclusion:**

In this cohort study of patients with mild stroke, autonomic dysfunction was highly prevalent and persisted six months after index stroke. Head-up tilt table test may be used in patients who tolerate active standing. Autonomic dysfunction should be recognized and handled in the early phase after stroke.

## Introduction

Stroke is one of the most common causes of death and the leading cause of long-term disability in the world [1]. Autonomic dysfunction (AD) is reported present in 25%-76% of patients with acute stroke [2–4]. Pathophysiological, damage to the insula cortex is associated with AD [5, 6], but practically any damage to the central network may potentially damage the intricate autonomic nervous system [7] and AD is therefore prevalent in many stroke patients. The central autonomic network is highly complex and involve telencephalic, diencephalic and brainstem structures [7]. The widespread, intrinsic connections make the autonomic nervous system (ANS) vulnerable to disturbances by comorbidities and commonly used medication in stroke patients such as diabetes, heart disease and beta-blocker-use [8]. It is still unclear whether AD precede stroke or is a consequence thereof. In a recent Framingham study [9], root mean successive squared difference (RMSSD) and standard deviation of successive normal beats (SDNN) was not associated with stroke risk which indicates that changes in RMSSD and SDNN do not precede stroke. Furthermore, observational studies have shown AD to be more prevalent in stroke patients compared to healthy controls independent of potential confounding covariables such as age and diabetes [10, 11].

AD assessed by conventional tests in the acute phase of stroke has been associated with progression in neurological symptoms, worse functional outcome, cardiovascular complications and increased mortality [4, 12–14] and AD is highly prevalent in chronic stroke patients [10]. One previous smaller study (*n* = 37) performed follow-up on the same stroke population including hemorrhagic stroke using conventional autonomic tests over time with recovery limited to one month [11]. Accordingly, changes in AD in stroke patients over time remain to be further resolved.

In the present cohort study, we aimed to examine whether head-up tilt table test (HUT) is tolerable in an acute stroke setting and describe AD in patients with mild ischemic stroke in the acute phase and after six months follow-up.

## Materials and methods

### Study design and setting

The present study was conducted on data from a prospective observational cohort study including consecutively recruited patients with acute ischemic stroke admitted to the Stroke Department, Copenhagen University Hospital—Rigshospitalet, Copenhagen, Denmark, from May 2015 to August 2016.

### Patient enrollment

Patients older than 18 years with acute ischemic stroke, who could be studied within seven days following the event were enrolled. Patients with hemorrhagic transformations were included.

Exclusion criteria were transitory ischemic attack (TIA); known brain disease such as multiple sclerosis, dementia, Parkinson disease or mental retardation based on ICD-10 diagnoses; known AD; fatal strokes or severe comorbidity with short life expectancy (months); decreased level of consciousness; hemorrhagic stroke and if study investigators judged the patient unable to complete the study protocol.

Due to logistic reasons only one patient could be enrolled at a time. If more than one candidate were admitted at the same time, the patient first admitted was enrolled.

Additional exclusion criteria for HUT were severe paresis and/or severe aphasia; lack of standing function and weight above 150 kg. In all autonomic tests beside HUT, patients with atrial fibrillation at examination time were excluded.

On admission, stroke severity and disability were assessed with National Institutes of Health Stroke Scale (NIHSS) and modified Ranking Scale (mRS). Besides brain CT scan, patients also had a brain magnetic resonance imaging (MRI) if possible. Based on standard stroke work-up the presumed stroke etiology was categorized according to criteria established by the Trial of Org 10,172 in Acute Stroke Treatment (TOAST).

### MRI and carotid ultrasound

The MRIs were performed with a Siemens Avanto 1.5 T Scanner with standard protocol including sagittal axial fluid attenuation inversion recovery, 3D T1, susceptibility-weighted imaging, and diffusion-weighted imaging. Lesions were described by a blinded neuroradiologist according to the STandards for ReportIng Vascular changes on nEuroimaging (STRIVE) [15]. Lesion sites were described using J. Wardlaw, University of Edinburgh imaging rating tool. Furthermore, a total small vessel disease (SVD) score was calculated (range 0–4) giving one point for each of the following characteristics: 1) non-acute lacuna 2) deep and periventricular white matter hyperintensity (Fazekas score 2–3) 3) microbleeds or 4) enlarged perivascular spaces in the basal ganglia [16].

The degree of atherosclerosis in the internal carotid arteries was assessed according to the Society of Radiologists in Ultrasound criteria [17]. The highest score of either left or right internal carotid artery was reported (Table 1).

**Table 1:** Baseline characteristics of all acute stroke patients at inclusion and divided by whether they completed in-hospital follow-up or not

	All (*N* = 91^1^)	With follow-up (*N* = 66^1^)	No follow-up (*N* = 25^1^)	*p*-value^2^
**Male (sex)**	52 (57%)	42 (64%)	10 (40%)	0.072
**Age**	66 (11)	66 (11)	67 (12)	0.855
**BMI**	27.6 (6.0)	27.3 (5.2)	28.4 (7.9)	0.943
**History of smoking**				
Current	26 (29%)	21 (32%)	5 (20%)	0.514
Ex. Smoker	34 (37%)	23 (35%)	11 (44%)	
Never-smoker	31 (34%)	22 (33%)	9 (36%)	
** Baseline mRS**	2 (1, 3)	2 (1, 3)	2 (1, 4)	0.403
** Baseline NIHSS**	2 (1, 4)	2 (1, 4)	2 (1, 3)	0.945
**Lesion site**				
Insula	9 (13%)	6 (12%)	3 (17%)	0.693
Brainstem	9 (14%)	8 (16%)	1 (5.9%)	0.427
**TOAST**				
Cardioembolic	13 (14%)	9 (14%)	4 (16%)	0.708
Large artery occlusion	10 (11%)	6 (9%)	4 (16%)	
Small vessel occlusion	23 (25%)	19 (29%)	4 (16%)	
Other etiology	1 (1%)	1 (2%)	0 (0%)	
Undetermined etiology	44 (48%)	31 (47%)	13 (52%)	
**Total SVD score**				
0	26 (32%)	18 (30%)	8 (38%)	0.796
1	17 (21%)	12 (20%)	5 (24%)	
2	18 (22%)	14 (23%)	4 (19%)	
3	18 (22%)	15 (25%)	3 (14%)	
4	3 (3.7%)	2 (3.3%)	1 (4.8%)	
Missing	9	5	4	
**Internal carotid atherosclerosis**				
None	13 (14%)	11 (17%)	2 (8%)	0.768
Mild	27 (30%)	20 (30%)	7 (28%)	
Moderate	34 (37%)	23 (35%)	11 (44%)	
Severe	12 (13%)	9 (14%)	3 (12%)	
**Comorbidities**				
Diabetes type I or II	19 (21%)	14 (21%)	5 (20%)	> 0.999
Hypertension	54 (59%)	38 (58%)	16 (64%)	0.751
History of stroke	15 (16%)	10 (15%)	5 (20%)	0.546
History of MI	10 (11%)	8 (12%)	2 (8%)	0.721
Atrial fibrillation	17 (19%)	11 (17%)	6 (24%)	0.547
Migraine	13 (14%)	10 (15%)	3 (12%)	> 0.999
Cancer	6 (7%)	5 (8%)	1 (4%)	> 0.999
**Medications**				
Beta blockers	34 (37%)	23 (35%)	11 (44%)	0.574
Calcium channel blockers	22 (24%)	17 (26%)	5 (20%)	0.765
RAS inhibitors	35 (38%)	25 (38%)	10 (40%)	> 0.999
Statins	65 (71%)	47 (71%)	18 (72%)	> 0.999

^1^Statistics presented: n (%); mean (SD); median (IQR)

^2^Between patients with and without follow-up. Statistical tests performed: Wilcoxon rank-sum test; Fisher's exact test; chi-square test of independence

*mRS* modified Ranking Scale, *NIHSS* National Institutes of Health Stroke Scale, *TOAST* Trial of ORG 10,172 in Acute Stroke Treatment, *MI* myocardial infarction, *RAS* Renin-angiotensin system

### Autonomic nervous system test

Measurement of blood pressure and heart rate were made with the Task Force® Monitor (CNSystems, Medizintechnik GmbH, Austria) non-invasively. The system monitored four-lead ECG, transthoracic impedance for calculation of stroke volume, beat-to-beat blood pressure by photoplethysmography on the middle finger with the hand at heart level and arm blood pressure cuff on the contralateral arm for calibration. All tests were performed within seven days after stroke with follow-up approximately six months after. Examination was conducted by trained study investigators at room temperature in noise free surroundings. Patients fasted for two hours prior to testing and were informed not to consume coffee, alcohol, smoke cigarettes, or use unnecessary medication. The procedure was conducted between 8 and 12AM and patients were thoroughly instructed in the procedure. During the actual procedures, communication was kept at a minimum to avoid interfere with the test results.

The patients rested for 10 min in the supine position followed by 10 min of baseline recording and drawing of blood samples for catecholamines from a cubital vein. The series of tests were then conducted as follows:

### The Valsalva maneuver

The Valsalva maneuver was performed in a sitting position with 15 s forced expirations in a manometer with 40 mmHg resistance. The maneuver was repeated until two replicable maneuvers were obtained. Repeated measurements where done, with intervals allowing blood pressure and heart rate to be stabilized.

### Heart rate response to deep breathing

The deep breathing test was conducted in a sitting position over six consecutive breathing cycles using five seconds for inhalations and exhalations, respectively. A practice session was done before the actual measurement.

### Active standing

Patients who were able then did an active standing test. The patients rested for three minutes in supine position after which by assistance they rose in a fluent movement and stood for three minutes without assistance before returning to supine position. The test was terminated if the patient developed symptoms like dizziness, nausea, headache, sweating, blackouts, altered mental status, or new onset of neurological symptoms.

### HUT

In patients who tolerated the active standing test, HUT was done following a period of 10 min supine rest, until heartbeat and blood pressure were stable. The examination was terminated if the patient developed symptoms as described under Active standing. The patients were placed with their feet on the footrest and with safety belts around the chest and abdomen. Patients were then tilted in a 60-degree angle for 25 min and monitored for tolerance, heart rate and blood pressure.

### Blood catecholamines

Concentrations of adrenaline and noradrenaline were determined at baseline after approximately 20 min of rest in supine position and after 15 min in the tilted position. Three glasses of nine milliliters of blood were drawn from the cubital vein at each sample. The samples were immediately stored at five °C for a maximum of one hour, centrifuged in 10 min with 2200G RCF at four °C, pipetted and then stored at -80 °C until analysis. The analysis was carried out by Diagnostic Department, Rigshospitalet, Glostrup, by radioimmunoassay using the validated commercial kit 2-CAT RIA, BA R-6500 (Labor Diagnostika Nord Gmbh (LDN), Nordhorn, Germany).

### Heart rate variability (HRV)

A continuous ECG was obtained during the procedure and the baseline recording after the initial supine rest was used for data analysis.

HRV analysis was done using the software program Kubios, version 2.0 [18]. Artifact- and ectopic free three minutes segments of the ECG were used for the analysis of RR intervals in stationary supine position.

Frequency analysis was performed using the autoregressive model and quantified in the following frequency bands: VLF (< 0.04 Hz), LF (0.04–0.15 Hz), and HF (0.15–0.40 Hz). The power was quantified by measuring the integral of the power spectral density curves of these bands and were expressed as both absolute power (ms2) and in normalized units (n.u.). The total spectral power was also reported. In the time domain, mean value of successive normal beats (mean (NN)), SDNN and the numerical difference between successive normal beats RMSSD were reported as recommended by the current guidelines [19].

### Autonomic dysfunction outcome variables quantification

Parasympathetic cardiovagal impairment was assessed and quantified by respiratory sinus arrythmia (RSA), sex adjusted abnormal Valsalva ratio (VR) and 30:15 ratio which were defined as following [20]:

- RSA was defined as mean difference between maximum RR-interval under expiration and minimum RR-interval under inspiration during six respiratory cycles.
- VR was defined as the maximum rate heart during the maneuver divided by the lowest heart rate obtained within 30 s after the maximum heart rate.
- 30:15 ratio was defined as the ratio of the maximum and minimum RR interval the 30th and 15th heartbeat during active standing test.

Abnormal results were defined as follows:

- RSA below age adjusted cut-of normal value (95th percentile) [21] or no heart rate response.
- VR below age and sex adjusted cut-of normal value (95th percentile) [21] or no heart rate response.
- 30:15 ratio < 1.0 [22].

Sympathetic vasomotor adrenergic impairment was defined as a persistent drop in systolic blood pressure (SBP) of > 20 mmHg or a diastolic blood pressure drop of > 10 mmHg [23] during HUT and by a lack of increase in blood pressure during phase IIb of the Valsalva maneuver. The adrenergic impairment of the Valsalva maneuver was graded according to the adrenergic score of Composite Autonomic Severity Score (CASS) [24].

Abnormal catecholamine response was defined as a smaller than a 60% increment from baseline during HUT [25].

### Definition of tolerance parameters in HUT

HUT tolerance was operationalized as following: 1) systolic blood pressure within 50 mmHg from baseline measurement 2) absence of cardiac asystole for more than three seconds 3) absence of syncope 4) and absence of new onset of neurological deficits.

### Statistical analysis

Continuous variables were quantified as mean (Standard Deviation (SD)) or median (Interquartile Range (IQR)). Categorical variables were presented as counts (%).

Change in autonomic dysfunction at follow-up was tested with McNemar's Chi-squared test with continuity correction for paired dichotomous variables. Wilcoxon signed-rank test was used to test for change in paired ordered variables and paired T test for continuous normal distributed variables over time. Change over time were calculated on patients with test available from both inclusion and follow-up. Finally, a subgroup analysis excluding patients with a prescription of a beta-blocker and/or a renin-angiotensin inhibitor and/or diabetes type 1 or 2 ICD-10 diagnosis at index stroke was done. All analysis was performed using R (The R Foundation, Vienna, AU) version 4.0.3 with P-value ≤ 0.05 considered as statistically significant.

## Results

### Patients

**Fig. 1:**
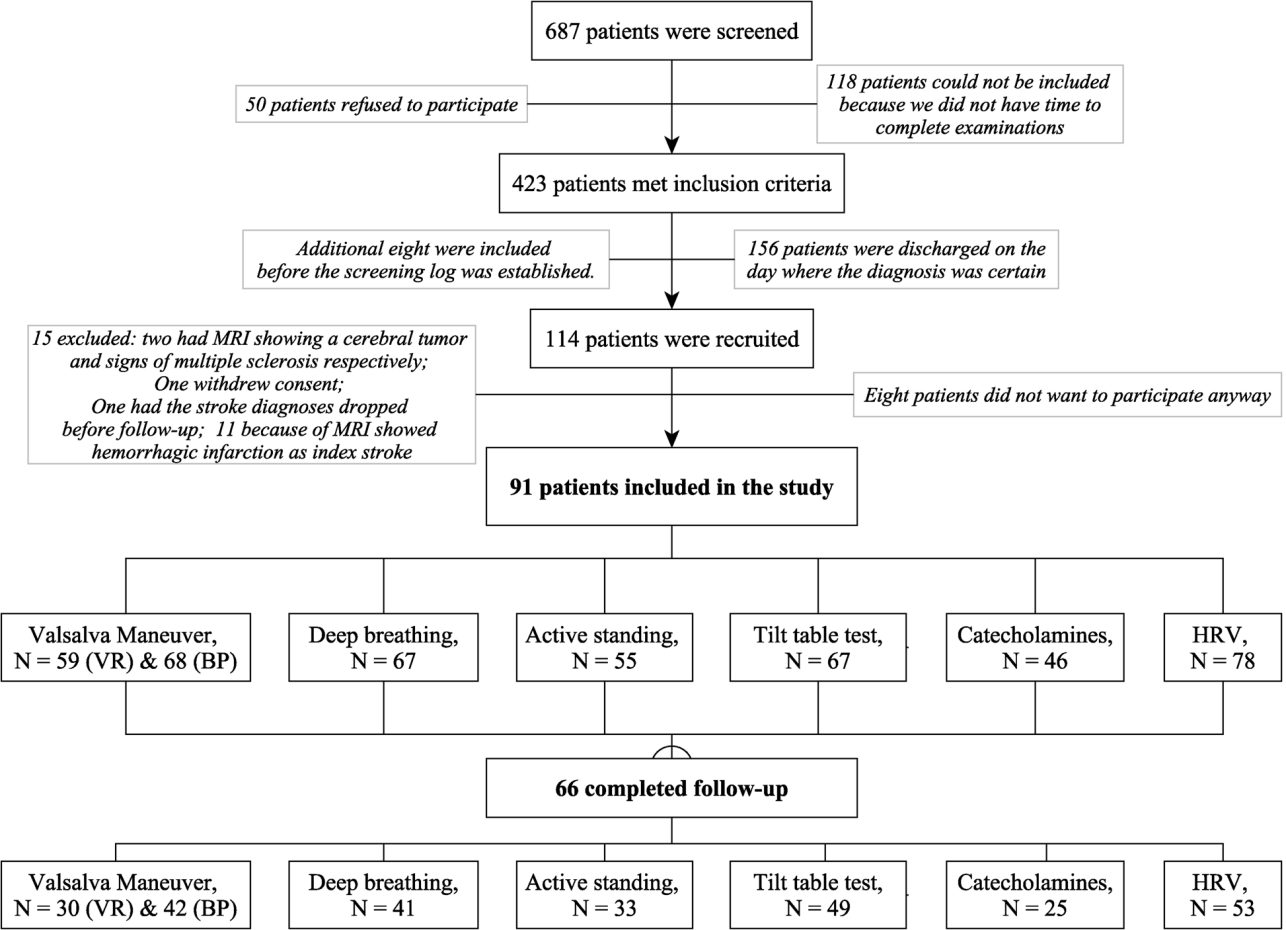
Overview over participant recruitment, testing and follow-up. VR = Valsalva Ratio; VM = Valsalva maneuver; BP = blood pressure; HRV = heart rate variability

The screening, inclusion and follow-up are illustrated in Fig. 1.

Baseline characteristics for all patients, patients with follow-up and patients without follow-up are shown in Table 1. Median (IQR) time until examination from index stroke date was 3 days (1, 4) and median (IQR) time until retesting at follow-up was 191 days (182, 200).

Baseline characteristics did not differ between patients who completed the follow-up and those who did not.

### Autonomic testing

**Fig. 2:**
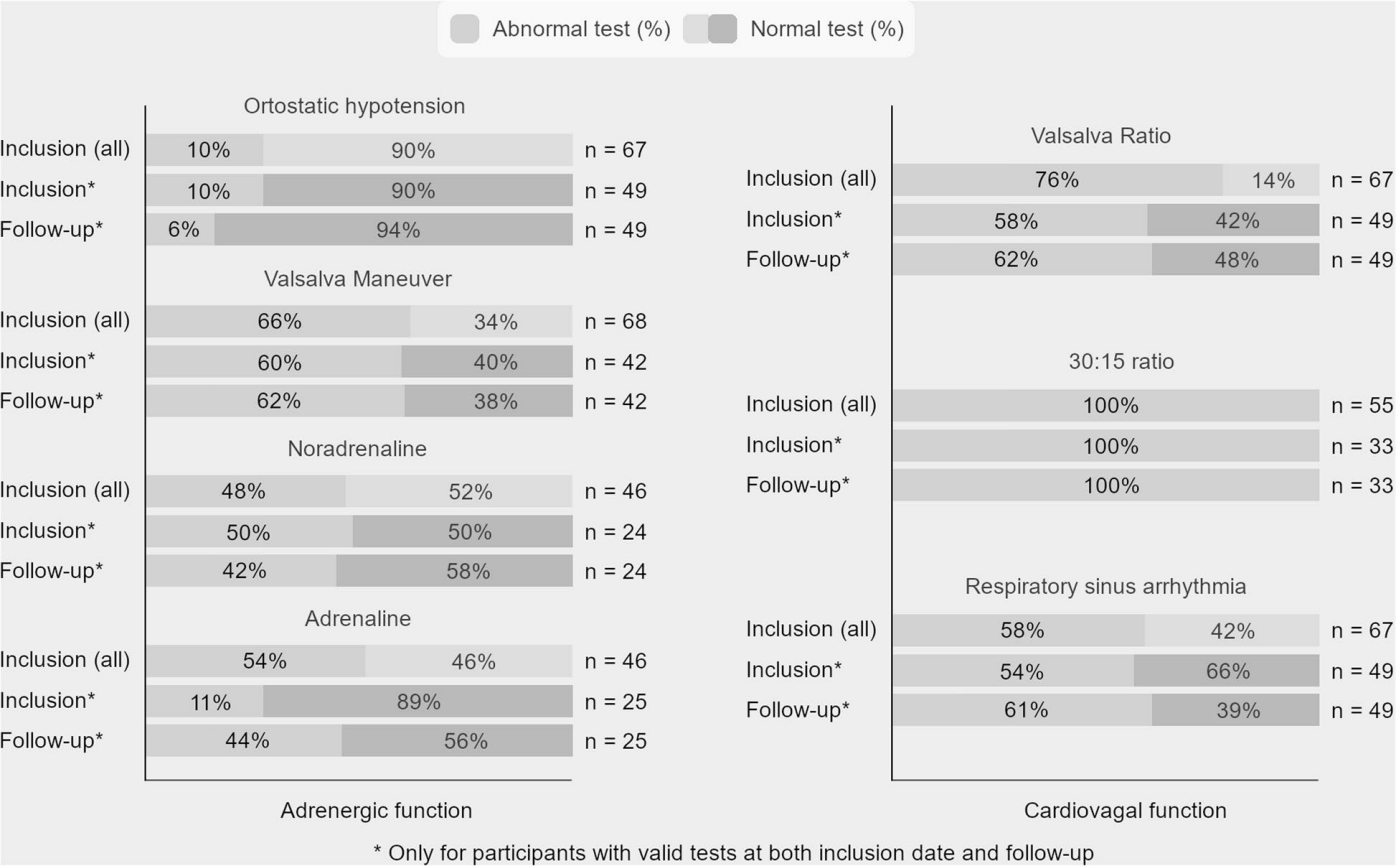
An overview of the results. Abnormal test (%) for all participants at inclusion date (Inclusion (all)), for participants with valid tests at inclusion date and follow-up (Inclusion* and Follow-up*)

An overview of the results is shown in Fig. 2. Results from all autonomic tests except HRV at inclusion are shown in Table 2, HRV in Table 3 and changes over time in Table 4.

**Table 2:** The prevalence of autonomic dysfunction (AD) at inclusion in patients with valid tests both at inclusion and at follow-up

	N	Mean (SD)	Number of patients with abnormal values
**Orthostatic hypotension**	67	-	7 (10%)
**Valsalva Ratio**	59	1.27 (0.20)	45 (76%)
**Abnormal BP to VM**	68	-	45 (66%)
Mild impairment		-	16 (24%)
Moderate impairment		-	13 (19%)
Severe impairment		-	16 (24%)
**RSA**	67	8.48 (5.69)	39 (58%)
**30:15 ratio**	55	0.90 (0.07)	55 (100%)
**Δ Noradrenaline** **[baseline] ng/ml**	46	1.49 (1.18) [2.35 (1.04)]	22 (48%)
**Δ Adrenaline [baseline] ng/ml**	46	0.0960 (0.111) [0.126 (0.0862)]	25 (54%)

*BP* blood pressure, *VM* Valsalva maneuver, *RSA* respiratory sinus arrythmia

**Table 3:** Heart rate variability (HRV) parameters at inclusion in patients with valid tests both at inclusion and at follow-up

*N* = 78	Mean NN (ms)	SDNN (ms)	RMSSD (ms)	PSD (ms^2^)	LF (ms^2^)	HF (ms^2^)	LF (n.u.)	HF (n.u.)	LF/HF
Mean (SD)	913 (138)	27 (1.94)	32 (29.4)	853 (1379)	347 (584)	411 (782)	52 (23.2)	48 (23.0)	1.93 (2.07)

*Mean NN* mean time between two successive heart beats, *SDNN* Standard deviation of the NN intervals, *RMSSD* root mean squared successive differences, *PSD* power spectral density (total power), *LF* low frequency domain, *HF* high frequency domain

**Table 4:** Autonomic dysfunction test over time for patients who completed inclusion and follow-up

	N^*^	**Inclusion**^**1**^	**Follow-up**^**1**^	**95% CI**	***p*-value**
**Orthostatic hypotension**	49	5 (10%)	3 (6%)	-	0.683
**Valsalva Ratio**	29	1.35 (0.22)	1.30 (0.20)	-0.12, 0.022	0.171
Abnormal	34	17 (58%)	20 (69%)	-	0.450
**Abnormal blood pressure to Valsalva maneuver**	42	25 (60%)	26 (62%)	-	0.850
**RSA**	39	8.8 (5.86)	7.8 (4.49)	-2.7, 0.45	0.152
Abnormal	41	22 (54%)	25 (61%)	-	0.547
**30:15 ratio**	33	0.89 (0.07)	0.90 (0.57)	-0.015, 0.042	0.347
Abnormal	33	33 (100%)	33 (100%)	-	1.0
**Δ Noradrenalin ng/ml (supine to tilt)**	24	1.47 (1.38)	1.41 (0.83)	-0.61, 0.50	0.833
Abnormal	24	12 (50%)	10 (42%)	-	0.724
**Δ Adrenalin ng/ml (supine to tilt)**	25	0.12 (0.13)	0.0928 (0.099)	-0.081, 0.033	0.395
Abnormal	25	7 (28%)	11 (44%)	-	0.134
**RMSSD**	53	21.9 (12.6, 42.1)	17.8 (12.1, 34.8)	-	0.400
**LF nu**	53	54 (24)	48 (23)	-12.3, 0.52	0.0707
**HF nu**	53	46 (24)	52 (23)	-0.49, 12.3	0.0696
**LF/HF**	53	0.99 (0.54, 3.64)	1.01 (0.44, 1.79)	**-**	0.0504
**Log(Total Power ms**^**2**^**)**	53	6.01 (1.25)	5.80 (1.15)	-0.56, 0.149	0.251^2^

^1^ Statistics presented: n (%), mean (SD), median (IQR)

^2^ Log transformed for normality assumption

*RSA* respiratory sinus arrythmia, *RMSSD* root mean squared successive differences, *LF* low frequency domain, *HF* high frequency domain

### Valsalva maneuver

A valid Valsalva maneuver measurement at enrollment was found in 59 out of the 91 patients (65%). Of the 32 non-valid measurements: 12 patients had extrasystoles; nine patients had atrial fibrillation; seven patients had no reason registered; two patients had technical issues and two patients could not cooperate to the examination.

Abnormal VR at inclusion was found in 45 patients (76%).

At follow-up 30 patients (55%) had valid measurements, and 21 patients (70%) had an abnormal VR value. No difference was found over time (*p* = 0.191).

A valid blood pressure response to the Valsalva maneuver measurement at enrollment was found in 68 out of the 91 patients (75%). Of the 23 non-valid measurements: 12 patients because of technical issues; six patients could not cooperate to the examination and five patients had a square-wave BP variant of the Valsalva maneuver.

Abnormal blood pressure response to the Valsalva maneuver at enrollment was found in 45 patients (66%) of which 23.5%, 19% and 23.5% had mild, moderate and severe impairment, respectively.

Forty two patients (62%) had valid measurements at follow-up, and of those 26 (62%) had an abnormal blood pressure response to the Valsalva maneuver. No change was found over time (*p* = 0.850).

### Heart rate response to deep breathing

A valid heart response to deep breathing measurement at enrollment was found in 67 out of the 91 patients (71%). 24 patients were excluded: nine for extrasystole; nine for atrial fibrillation; four due to technical issues and two due to lack of cooperation.

Abnormal RSA at inclusion was found in 39 patients (58%).

At follow-up 41 patients (61%) had valid measurements, of whom 22 (54%) had an abnormal RSA value. No change was found over time (*p* value = 0.752).

### Active standing

Results could be obtained in 55 of 91 patients (60%) as 18 patients did not complete the examination, seven had atrial fibrillation; four had too much noise in their ECG-curve in relation to the 30:15 ratio; four were excluded due to technical issues, three had extrasystoles and two patients were excluded because they developed dizziness.

Abnormal 30:15 ratio at inclusion was found in 55 patients (100%).

At follow-up 33 patients (60%) had valid measurements, of whom 33 patients (100%) having an abnormal 30:15 ratio. No change was found over time (*p*-value = 0.347).

### HUT

HUT was completed in 67 of 91 patients (74%). 24 did not complete testing: 22 fulfilled the exclusion criteria for HUT and two patients were excluded because they developed symptoms during active standing.

Orthostatic hypotension at inclusion was found in 7 patients (10%).

At follow-up, 49 patients (73%) had valid measurements and those three patients (6%) had orthostatic hypotension. No change was found over time (*p*-value = 0.683).

The examination was terminated in 11 out of 67 patients (15%) at enrollment according to the tolerability criteria. Seven patients because they had a blood pressure fall exceeding 50 mmHg and four patients because they developed symptoms such as dizziness, general malaise, or visual obscurations. No patients developed asystole or progression in neurological symptoms. Median (IQR) time until termination was 20 min (16, 22).

At follow-up, the examination was terminated on 5 out of 49 patients (10%). Three had a blood pressure fall exceeding 50 mm Hg and two patients developed severe symptoms (visual obscurations and fainting). No patients had asystole or onset of new/worsening of neurological symptoms. Median (IQR) time until termination at follow-up was 17 min (11, 21).

### Catecholamines

Valid catecholamine measurements at inclusion during both supine rest and HUT could be obtained in 46 of the 91 patients (51%). In 15 patients (33%) the increment in plasma adrenaline after 15 min of HUT was less than 60%.

At follow-up 25 patients (54%) had valid measurements. No difference in the increment of plasma adrenaline was found over time (*p*-value = 0.395).

In 22 patients (48%) at inclusion, the increment in plasma noradrenaline upon 15 min HUT was less than 60%. At follow-up 24 patients (52%) had valid measurements. No difference in the increment of plasma noradrenaline was found over time (*p*-value = 0.833).

### HRV

A valid ECG recording could be obtained in 78 out of 91 patients (86%)—nine had atrial fibrillation and four had an excess of extrasystoles. Results from inclusion can be viewed in Table 3.

At follow-up 53 patients (68%) had valid measurements. No changes over time were found.

### Lesion sites

Of the 7 patients at inclusion with orthostatic hypotension on HUT, none had lesions in either insula or in the brainstem. We found no association between orthostatic hypotension and cerebral SVD.

### Subanalysis

In a subgroup analysis excluding patients with prescription of either a beta-blocker or renin-angiotensin inhibitor at index stroke and/or a diabetes mellitus type 1 or 2 ICD-10 diagnosis, autonomic dysfunction was still highly prevalent and varied from 4/29 (14%) patients with orthostatic hypotension and 25/25 (100%) patients with abnormal 30:15 ratio at index stroke. The calculations were done for those patients who had valid measurements at index stroke.

## Discussion

To our knowledge this is the largest study assessing AD in ischemic stroke patients close to the event and with six months follow-up. We found the procedures to be tolerated in both the acute and chronic phase of stroke. However, HUT was only performed in patients who tolerated active standing and still one patient fainted in the study, so HUT should still be performed with caution with these patients.

In this prospective cohort study consisting of 91 mild acute ischemic stroke patients, we found that the prevalence of AD ranged widely from 10%-100% depending on the autonomic test. Impairments on the cardiovagal tests were more prevalent than on the sympathetic vasomotor and the impairments did not improve after a recovery period of six months.

### Pathophysiology of stroke induced autonomic dysfunction

As previously stated, the pathophysiology of AD is very complex involving widespread connections throughout the human body [8]. Extrainsular brain lesions have been reported to cause AD [10]. Insula insults, albeit an important brain structure for the autonomic nervous system, [5, 6] cannot explain entirely why AD occur in many stroke patients. In our study none of the patients with orthostatic hypotension had lesions involving insula. A strict segmental understanding of the autonomic nervous system is therefore likely not feasible. It seems as in other brain functions that the autonomic nervous system should be viewed from a network perspective and that disturbances in the autonomic nervous system network, regardless of the lesion site, cause alterations in the homeostatic regulatory functions [8]. Despite prevalent AD in stroke patients, it remains difficult to fully resolve the confounding impact from comorbidities and medication-use in stroke populations.

### Autonomic dysfunction in stroke patients

Our results are in accordance with a previous observational study by Xiong et al. [10] reporting a high prevalence of AD in both an acute (N 34) and a chronic stroke population (N 60, six months after index stroke) assessed by Ewing’s autonomic test battery. Xiong et al. [10] also found the cardiovagal impairments to be most prevalent. Our results are also in accordance with a prospective observational cohort study by Pandian et al. [11] reporting no change over time in autonomic dysfunction assessed by HUT, Valsalva maneuver or heart rate response to deep breathing one month after index (N 37). In the present study, we followed the patients six months after index stroke and found no change in the autonomic test parameters. However, like Xiong et al. [10], our results are in discordance with the findings of Pandian et al. [11] reporting predominantly changes in the sympathetic parameters.

Since there is a lack of well-established normal values for HRV [19], we cannot report the number of patients with abnormal values, but previous studies have shown alterations in HRV parameters in the acute phase of ischemic stroke [10, 26, 27]. However, only few studies have included follow-up and reported changes over time. In the prospective cohort studies by Korpelainen et al. [28, 29] (N 31 and 46) no changes were detected in the HRV parameters compared to healthy controls after six months follow-up. In the present study we found borderline-significant reduction in the LF/HF ratio implying more sympathetic activity in the acute phase of stroke.

### HUT in stroke patients

HUT is rarely used in an acute stroke setting and in Denmark it is directly advised against. A clinical concern of reducing blood flow to the damaged brain area in the upright posture because of impaired cerebral autoregulation may explain this cautious approach [30, 31]. The dynamic changes of cerebral autoregulation under various pathophysiological conditions are complex though and not fully understood [32].

Previous studies with small numbers of patients (N 12–36) have suggested that HUT is well-tolerated in acute stroke patients, these studies included both ischemic and hemorrhagic strokes and one study included only cerebellar infarctions [33–36]. Our results from 67 mild ischemic stroke patients who tolerated active standing, undergoing HUT in an acute stroke setting are in accordance with these studies. No evidence of any clinical harm performing HUT has emerged and HUT has been shown to have low bias to potential confounding covariables such as medication and comorbidities [37] and is thereby applicable in acute stroke patients where comorbidities and polymedication with for example beta blockers is highly prevalent. Ischemic stroke patients tolerating early mobilization should therefor most likely also tolerate HUT.

Our results add to the evidence that autonomic dysfunction is highly prevalent in ischemic stroke patients and persists six months after index stroke. It is important to diagnose orthostatic hypotension in stroke patients, as it may hinder necessary rehabilitation and may lead to increased morbidity owing to an increased risk of falls and in worse case syncope, in patients that are already frail. This should be taken into consideration in the everyday clinical work with stroke patients, where the negative consequences of AD on the functional status and on the cardiovascular and immune system must be handled in the early phase after stroke. A similar clinical approach is already implemented for neurodegenerative diseases such as Parkinson disease.

### Limitations

The cohort was from a single-center, and only included patients with milder stroke and may thus represent selection bias although if prevalent in mild stroke patients, it is likely that it is also prevalent in more moderate-severe stroke patients. Age and gender adjusted normal values were used for all tests except HRV, because it is very difficult to find a representative control group due to the substantial number of co-morbidities in the stroke patients. The lack of an age and sex matched healthy control group may limit comparability. However, the reason to have a control group is to study the independent variable (stroke) without confounding conditions on autonomic dysfunction. Comparing a stroke population to a healthy control group would differ on so many confounding variables anyway beside the stroke, since a considerable number of known and unknown confounders are associated with having a stroke including medication and comorbidities. The included stroke patients had potential confounders such as diabetes and usage of medication for cardiovascular diseases which could imply that some patients had autonomic dysfunction prior to index stroke. Not including these patients would lead to selection bias since it is well known that ischemic stroke patients often have many comorbidities. In the subgroup analysis, excluding patients treated with beta-blockers, renin-angiotensin inhibitors and/or having diabetes mellitus, autonomic dysfunction was still highly prevalent. No patients were in treatment with dopamine agonists which limits bias from neurodegenerative diseases such as Parkinson. Due to the sample size, we cannot make conclusions regarding the importance of lesion site or load of cerebral SVD. Follow-up rate was relatively low but was expected in an elderly stroke cohort with physical and cognitive sequelae. Some patients had invalid measurements or could not complete the entire work-up. This is a well-known challenge in acute stroke patients characterized by reduced physical function, cognitive challenges and high degree of fatigue.

## Conclusion

In this cohort study of patients with mild stroke, HUT may be used in patients who tolerate active standing with caution since one patient fainted in the study. AD was highly prevalent and persistent six months after index stroke. Screening for AD should be recognized and handled as part of the stroke patient trajectories in the early phase after stroke.

## Data Availability

Data available upon reasonable requests to author Sofie Amalie Simonsen, however, as per Danish law approval from the Danish Patient Safety Authority and the Greater Capital Region of Copenhagen’s Data Safety Board might be required.

## References

[1] GBD 2016 Stroke Collaborators. Global, regional, and national burden of stroke, 1990–2016: a systematic analysis for the Global Burden of Disease Study 2016. Lancet Neurol. 2019;18(5):439–458. doi:10.1016/S1474-4422(19)30034-1 10.1016/S1474-4422(19)30034-1 PMC6494974 30871944

[2] Nayani S, Sreedharan SE, Namboodiri N, Sarma PS, Sylaja PN (2016). Autonomic dysfunction in first ever ischemic stroke: Prevalence, predictors and short term neurovascular outcome. Clin Neurol Neurosurg.

[3] Xiong L, Leung H, Chen XY (2012). Preliminary findings of the effects of autonomic dysfunction on functional outcome after acute ischemic stroke. Clin Neurol Neurosurg.

[4] Xiong L, Tian G, Leung H (2018). Autonomic Dysfunction Predicts Clinical Outcomes After Acute Ischemic Stroke: A Prospective Observational Study. Stroke.

[5] Oppenheimer SM, Gelb A, Girvin JP, Hachinski VC (1992). Cardiovascular effects of human insular cortex stimulation. Neurology.

[6] de Morree HM, Rutten GJ, Szabó BM, Sitskoorn MM, Kop WJ (2016). Effects of Insula Resection on Autonomic Nervous System Activity. J Neurosurg Anesthesiol.

[7] Mo J, Huang L, Peng J, Ocak U, Zhang J, Zhang JH (2019). Autonomic Disturbances in Acute Cerebrovascular Disease. Neurosci Bull.

[8] Kaufmann H, Norcliffe-Kaufmann L, Palma JA (2020). Baroreflex Dysfunction. N Engl J Med.

[9] Weinstein G, Davis-Plourde K, Beiser AS, Seshadri S (2021). Autonomic Imbalance and Risk of Dementia and Stroke: The Framingham Study. Stroke.

[10] Xiong L, Leung HH, Chen XY (2013). Comprehensive assessment for autonomic dysfunction in different phases after ischemic stroke. Int J Stroke.

[11] Pandian JD, Dalton K, Scott J, Read SJ, Henderson RD (2011). Cardiovascular autonomic dysfunction in acute stroke. Int J Stroke.

[12] Ha SY, Park KM, Park J, Kim SE, Lee BI, Shin KJ (2018). Autonomic function test in progressive lacunar infarction. Acta Neurol Scand.

[13] Colivicchi F, Bassi A, Santini M, Caltagirone C (2005). Prognostic implications of right-sided insular damage, cardiac autonomic derangement, and arrhythmias after acute ischemic stroke. Stroke.

[14] McLaren A, Kerr S, Allan L (2005). Autonomic function is impaired in elderly stroke survivors. Stroke.

[15] Wardlaw JM, Smith EE, Biessels GJ (2013). Neuroimaging standards for research into small vessel disease and its contribution to ageing and neurodegeneration. Lancet Neurol.

[16] Staals J, Makin SD, Doubal FN, Dennis MS, Wardlaw JM (2014). Stroke subtype, vascular risk factors, and total MRI brain small-vessel disease burden. Neurology.

[17] Grant EG, Benson CB, Moneta GL (2003). Carotid artery stenosis: gray-scale and Doppler US diagnosis–Society of Radiologists in Ultrasound Consensus Conference. Radiology.

[18] Tarvainen MP, Niskanen JP, Lipponen JA, Ranta-Aho PO, Karjalainen PA (2014). Kubios HRV–heart rate variability analysis software. Comput Methods Programs Biomed.

[19] Heart rate variability. Standards of measurement, physiological interpretation, and clinical use. Task Force of the European Society of Cardiology and the North American Society of Pacing and Electrophysiology. Eur Heart J. 1996;17(3):354–381. 8737210

[20] Ewing DJ, Campbell IW, Clarke BF (1980). Assessment of cardiovascular effects in diabetic autonomic neuropathy and prognostic implications. Ann Intern Med.

[21] Low PA, Denq JC, Opfer-Gehrking TL, Dyck PJ, O'Brien PC, Slezak JM (1997). Effect of age and gender on sudomotor and cardiovagal function and blood pressure response to tilt in normal subjects. Muscle Nerve.

[22] Gautschy B, Weidmann P, Gnädinger MP (1986). Autonomic function tests as related to age and gender in normal man. Klin Wochenschr.

[23] Freeman R, Wieling W, Axelrod FB (2011). Consensus statement on the definition of orthostatic hypotension, neurally mediated syncope and the postural tachycardia syndrome. Clin Auton Res.

[24] Low PA (1993). Composite autonomic scoring scale for laboratory quantification of generalized autonomic failure. Mayo Clin Proc.

[25] Goldstein DS, Cheshire WP (2018). Roles of catechol neurochemistry in autonomic function testing. Clin Auton Res.

[26] Kanai M, Kubo H, Kitamura Y (2015). Difference in autonomic nervous activity in different subtypes of noncardioembolic ischemic stroke. Int J Cardiol.

[27] Lakusić N, Mahović D, Babić T, Sporis D (2003). Promjene autonomne kontrole srcanog rada u bolesnika s preboljelim ishemijskim mozdanim udarom [Changes in autonomic control of heart rate after ischemic cerebral stroke]. Acta Med Croatica.

[28] Korpelainen JT, Sotaniemi KA, Huikuri HV, Myllyä VV (1996). Abnormal heart rate variability as a manifestation of autonomic dysfunction in hemispheric brain infarction. Stroke.

[29] Korpelainen JT, Sotaniemi KA, Mäkikallio A, Huikuri HV, Myllylä VV (1999). Dynamic behavior of heart rate in ischemic stroke. Stroke.

[30] Treger I, Shafir O, Keren O, Ring H (2006). Orthostatic hypotension and cerebral blood flow velocity in the rehabilitation of stroke patients. Int J Rehabil Res.

[31] Intharakham K, Beishon L, Panerai RB, Haunton VJ, Robinson TG (2019). Assessment of cerebral autoregulation in stroke: A systematic review and meta-analysis of studies at rest. J Cereb Blood Flow Metab.

[32] Xiong L, Liu X, Shang T (2017). Impaired cerebral autoregulation: measurement and application to stroke. J Neurol Neurosurg Psychiatry.

[33] Baltz MJ, Lietz HL, Sausser IT, Kalpakjian C, Brown D (2013). Tolerance of a standing tilt table protocol by patients an inpatient stroke unit setting: a pilot study. J Neurol Phys Ther.

[34] Miyake T, Nakamura T, Kouda K, et al. Carotid blood flow, cardiovascular and endocrine responses during head-up tilt in patients with acute cerebrovascular diseases. Springerplus. 2014;3:191. Published 2014 Apr 16. doi:10.1186/2193-1801-3-191 10.1186/2193-1801-3-191 PMC4008730 24808998

[35] Enishi K, Tajima F, Akimoto H, Mita R (2004). Initial drop of blood pressure during head-up tilt in patients with cerebrovascular accidents. Environ Health Prev Med.

[36] Kim HA, Lee H (2016). Orthostatic hypotension in acute cerebellar infarction. J Neurol.

[37] Paul B, Gieroba Z, Mangoni AA (2007). Influence of comorbidities and medication use on tilt table test outcome in elderly patients. Pacing Clin Electrophysiol.

[38] Mol A, Bui Hoang PTS, Sharmin S (2019). Orthostatic Hypotension and Falls in Older Adults: A Systematic Review and Meta-analysis. J Am Med Dir Assoc.

